# Draft Genome Sequence of *Actibacterium mucosum* KCTC 23349, a Marine Alphaproteobacterium with Complex Ionic Requirements Isolated from Mediterranean Seawater at Malvarrosa Beach, Valencia, Spain

**DOI:** 10.1128/genomeA.00486-14

**Published:** 2014-05-22

**Authors:** David R. Arahal, Zongze Shao, Qiliang Lai, María J. Pujalte

**Affiliations:** a Departamento de Microbiología y Ecología and Colección Española de Cultivos Tipo (CECT), Universidad de Valencia, Valencia, Spain; b Key Laboratory of Marine Genetic Resources, Third Institute of Oceanography, State Oceanic Administration, Xiamen, China

## Abstract

Strain R46 (CECT 7668; KCTC 23349), a nomenclatural type of *Actibacterium mucosum*, was isolated from surface seawater collected at Malvarrosa Beach (Valencia, Spain) in July 2008. The draft genome sequence of strain R46 (approximately 3.72 Mbp) contains 22 scaffolds and 3,619 protein-encoding genes, with a G+C content of 60.8 mol%.

## GENOME ANNOUNCEMENT

The *Roseobacter* clade is the largest group in the family *Rhodobacteraceae* and the one that continues to grow at the fastest rate (1). It is dominated by species of marine origin, chemoorganoheterotrophic and nonfermentative. Most of these bacteria require Na ions (or sometimes combined marine salts) for growth, although other metabolic abilities and physiological traits are also known. Their outstanding role in marine ecosystems has been summarized and reviewed often and has led to advances in genomics (2–4). Genome sequences and features of members of the *Roseobacter* group are reported and updated at the site Roseobase-Genomic Resource for Marine Roseobacters (http://www.roseobase.org/index.html).

Strain R46 was isolated from surface seawater collected at Malvarrosa Beach (Valencia, Spain, 39°28′29″N, 0°19′23″W; temperature 25°C; pH 8.0) in July 2008, plated directly on Marine R2A agar, and incubated at 26°C for 8 days. Subsequent characterization permitted its proposal as the type strain of a novel genus and species, *Actibacterium mucosum*, which was made publicly available as CECT 7668 and KCTC 23349 (5).

Strain R46 is a strictly aerobic chemoorganotroph with complex ionic requirements. Cells do not accumulate poly-β-hydroxybutyrate and contain phosphatidylglycerol, an unidentified aminolipid, and a lipid as major polar lipids. Q10 is the predominant quinone and C_18:1_ *ω*7*c* is the major fatty acid. It is oxidase and catalase positive. It is not pigmented and its cells are ovoid to rod-shaped and non-motile. It is able to grow on defined medium with several carbohydrates, organic acids, and amino acids as sole carbon and energy sources (5).

Genome sequencing of strain R46 was done using the Miseq system by Shanghai Majorbio Bio-pharm Technology Co., Ltd. (Shanghai, China). A total of 4,187,984 reads (clean pair-reads: 2,083,937×2; clean single-reads: 20,110) were assembled using Newbler 2.6. A draft genome of 22 scaffolds over 1,100 bp in size was obtained. Automatic gene annotation was carried out by the NCBI Prokaryotic Genomes Automatic Annotation Pipeline (PGAAP) (http://www.ncbi.nlm.nih.gov/genomes/static/Pipeline.html) (6). The genome of strain R46 was 3,715,337 bp in length (336-fold coverage), presented a high G+C content (60.8%), showed a coding density of 91.0%, and contained 3,619 protein coding sequences and 41 tRNA genes. A total of 1,931 proteins could be assigned to the cluster of orthologous groups (COG) families (7).

Genome annotation and analysis were also done using the RAST server (http://rast.nmpdr.org/) (8). Eleven genes (*sox*) for sulfur oxidation were found in the annotations. Thus, chemolithotrophy upon sulfur compounds such as sulfite and thiosulfate might be possible. Similarly, carbon monoxide oxidation also seems possible according to the seven annotations received. The presence of ABC chains of dimethyl sulfoxide reductase was also noticed.

### Nucleotide sequence accession number.

This whole-genome shotgun project has been deposited at DDBJ/ENA/GenBank under accession no. JFKE00000000. The version described in this paper is the first version.

## References

[1] Pujalte MJ Lucena T Ruvira MA Arahal DR Macián MC The family *Rhodobacteraceae*. *In* Rosenberg E DeLong EF Stackebrandt E Lory S Thompson F (ed), The prokaryotes—alphaproteobacteria and betaproteobacteria, 4th ed, vol 8, in press Springer Verlag, Berlin, Germany. 10.1007/978-3-642-30197-1_377

[2] Wagner-Döbler I Biebl H 2006 Environmental biology of the marine *Roseobacter* lineage. Annu. Rev. Microbiol. 60:255–280. 10.1146/annurev.micro.60.080805.142115 16719716

[3] Brinkhoff T Giebel H-A Simon M 2008 Diversity, ecology, and genomics of the *Roseobacter* clade: a short overview. Arch. Microbiol. 189:531–539. 10.1007/s00203-008-0353-y 18253713

[4] Buchan A González JM 2010 Roseobacter, p 1335–1343 *In* Timmis KN McGenity T Van Der Meer J De Lorenzo V (ed), Handbook of hydrocarbon and lipid microbiology. Springer Verlag, Berlin, Germany

[5] Lucena T Ruvira MA Garay E Macián MC Arahal DR Pujalte MJ 2012 *Actibacterium mucosum* gen. nov., sp. nov., a marine alphaproteobacterium from Mediterranean seawater. Int. J. Syst. Evol. Microbiol. 62:2858–2864. 10.1099/ijs.0.038026-0 22228661

[6] Angiuoli SV Gussman A Klimke W Cochrane G Field D Garrity G Kodira CD Kyrpides N Madupu R Markowitz V Tatusova T Thomson N White O 2008 Toward an online repository of Standard Operating Procedures (SOPs) for (meta)genomic annotation. Omics 12:137–141. 10.1089/omi.2008.0017 18416670 PMC3196215

[7] Tatusov RL Galperin MY Natale DA Koonin EV 2000 The COG database: a tool for genome-scale analysis of protein functions and evolution. Nucleic Acids Res. 28:33–36. 10.1093/nar/28.1.33 10592175 PMC102395

[8] Aziz RK Bartels D Best AA DeJongh M Disz T Edwards RA Formsma K Gerdes S Glass EM Kubal M Meyer F Olsen GJ Olson R Osterman AL Overbeek RA McNeil LK Paarmann D Paczian T Parrello B Pusch GD Reich C Stevens R Vassieva O Vonstein V Wilke A Zagnitko O 2008 The RAST server: Rapid Annotations using Subsystems Technology. BMC Genomics 9:75. 10.1186/1471-2164-9-75 18261238 PMC2265698

